# How medical students co-regulate their learning in clinical clerkships: a social network study

**DOI:** 10.1186/s12909-022-03259-0

**Published:** 2022-03-21

**Authors:** Derk Bransen, Erik W. Driessen, Dominique M. A. Sluijsmans, Marjan J. B. Govaerts

**Affiliations:** 1 School of Health Professions Education, Maastricht University, Maastricht, the Netherlands; 2 Department of Educational Development and Research, Faculty of Health, Medicine and Life Science, Maastricht University, Maastricht, the Netherlands; 3 Rotterdam University of Applied Sciences, Rotterdam, the Netherlands; 4 Department of Educational Development and Research, Faculty of Health, Medicine and Life Science, Maastricht University, Maastricht, the Netherlands

**Keywords:** Self-regulated learning, Co-regulated learning, Social network study, Clinical clerkships, Medical education

## Abstract

**Background:**

Self-regulated learning is a key competence to engage in lifelong learning. Research increasingly acknowledges that medical students in clerkships need others to regulate their learning. The concept of “co-regulated learning” captures this act of regulating one’s learning by interacting with others. To effectively cultivate such skills in students, we need to increase our understanding of co-regulated learning. This study aimed to identify the purposes for which students in different phases of clinical training engage others in their networks to regulate their learning.

**Methods:**

In this social network study, we administered a questionnaire to 403 medical students during clinical clerkships (65.5% response rate). The questionnaire probed into the composition of students’ co-regulatory networks and the purpose for which they engaged others in specified self-regulated learning activities. We calculated the proportion of students that engaged others in their networks for each regulatory activity. Additionally, we conducted ANOVAs to examine whether first-, second-, and third-year students differed in how they used their networks to support self-regulation.

**Results:**

Students used others within their co-regulatory networks to support a range of self-regulated learning activities. Whom students engaged, and the purpose of engagement, seemed to shift as students progressed through clinical training. Over time, the proportion of students engaging workplace supervisors to discuss learning goals, learning strategies, self-reflections and self-evaluations increased, whereas the proportion of students engaging peers to discuss learning strategies and how to work on learning goals in the workplace decreased. Of all purposes for which students engaged others measured, discussing self-reflections and self-evaluations were consistently among the ones most frequently mentioned.

**Conclusions:**

Results reinforce the notion that medical students’ regulation of learning is grounded in social interactions within co-regulatory networks students construct during clerkships. Findings elucidate the extent to which students enact self-regulatory learning within their co-regulatory networks and how their co-regulatory learning behaviors develop over time. Explicating the relevance of interactions within co-regulatory networks might help students and supervisors to purposefully engage in meaningful co-regulatory interactions. Additionally, co-regulatory interactions may assist students in regulating their learning in clinical workplaces as well as in honing their self-regulated learning skills.

**Supplementary Information:**

The online version contains supplementary material available at 10.1186/s12909-022-03259-0.

## Background

Self-regulated learning (SRL) is a key competence that physicians need to be able to engage in lifelong learning for high-quality care [1]. Although many medical curricula aim to foster the development of SRL skills, students often struggle to regulate their learning in the complex and dynamic clinical workplace [2]. Medical education research increasingly conceptualizes SRL as socially embedded activities, acknowledging that learners’ regulatory learning processes and activities are influenced by interactions with others within a particular setting [3–5]. As such, the notion of SRL is inextricably linked and intertwined with the concept of co-regulated learning (CRL), which specifies that learners’ social interactions with others in their environment mediate how they regulate their cognitions, behaviors, and motivation [6–8]. In clinical settings, CRL takes shape when medical students interact with peers, residents, physicians, or any other individual within their network to address activities, struggles, and considerations regarding the regulation of their learning. The networks in which interactions with meaningful others concern, influence, and contribute to students’ self-regulation can be conceptualized as *co-regulatory networks* that may pose obstacles to students’ regulatory learning, while also providing affordances [9].

Previous research has suggested that learners can have different reasons for engaging others in their co-regulatory networks. Pediatric residents, for instance, may interact with their supervisors with the aim to pursue learning goals [10], whereas surgical residents may enlist the help of supervisors to monitor their performance during surgery [11]. Moreover, such co-regulatory learning behaviors and activities seem to evolve as learners progress through training, with junior medical students mostly engaging their peers in co-regulatory endeavors and their more advanced counterparts preferring to involve residents and physicians [5, 12]. Although the importance of interactions with others to students’ regulation of learning is well-understood, network theories may offer more elaborated insights into the role learners’ co-regulatory networks play in regulating their learning in clinical settings. According to social network theory, the way individuals are embedded in their social connections influences their behavior [13]. Moreover, networks are structures consisting of actors (individuals) and links between these actors (ties) that capture, for example, the focus and patterns of communication [14]. To understand the importance of relationships in medical education and their influence on educational processes and outcomes, social network analysis (SNA) has proven crucial. Indeed, previous SNAs have revealed that the relationships medical students maintained predicted their learning outcomes [15], that medical students tend to select friends of the same sex and ethnicity [16], and that residents who were close to others in their networks tended to have higher degrees of personal accomplishment [17]. These findings highlight the potential of social network perspectives to investigate how students’ regulation of learning is embedded in the relationships they build and maintain during their clerkships.

Notwithstanding this, studies that have explored students’ networks for CRL have only rarely adopted a social network perspective. In our recent study, however, we did focus on the relationships between students’ co-regulatory network characteristics and their SRL and found that the frequency with which students engaged others in their co-regulatory networks and their self-reported SRL proficiency were positively and significantly related [18]. Yet, we did not clarify the specific regulatory purposes for which students engaged others in their networks. Hence, there is at present a paucity of information on medical students’ co-regulatory networks and how they use these networks to regulate their learning. In a bid to fill this gap, this study investigated students’ co-regulatory networks in clinical settings. More specifically, we aimed to examine the purposes for which medical students in different phases of clinical training engaged others in their co-regulatory networks to regulate their learning.

## Methods

We conducted a cross-sectional questionnaire study involving medical students in clinical clerkships in the period between November 2019 and February 2020. We collected data on both the structure of students’ co-regulatory networks and the focus of their interactions within these networks. We were particularly interested in the purposes for which students engaged others in their efforts to co-regulate learning during clerkships. We drew on social network analysis techniques, because it allowed us to examine whom students’ engage with, and what the communication focuses on. As such, the social network perspective and associated techniques are suited to provide information about our research aim. We provide a more detailed description of how we drew on SNA in the remainder of this method section.

### Setting

This study was set in the master’s program in medicine at Maastricht University, the Netherlands. Underpinned by the principles of competency-based medical education, this program has the roles of the Canadian Medical Education Directives for Specialists (CanMEDS) as its overarching assessment framework [19]. In the course of the program which spans three years of clinical training, students rotate through 8- to 18-week clerkships in an academic hospital and affiliated teaching hospitals. As such, their learning is mainly workplace-based. To support their SRL, students are assigned a mentor and workplace supervisor, and are required to compose an e-portfolio. For each clerkship, they set learning goals and formulate learning plans, which they consequently discuss with their supervisor and mentor [20]. After each clerkship, they evaluate and reflect on their learning together with their mentor, based on which they formulate new learning goals. These and other assessment requirements, including regular educational meetings, encourage students to follow the SRL cycle systematically.

### Participants and data collection

Students who were in one of the following mandatory clerkships were considered eligible for participation in our study: healthcare participation (HELP), surgery, internal medicine, mother and child, neurosciences, family and social medicine (*N* = 615). We approached students during educational meetings and invited them to participate in the study. To this end, the first author (DB) visited 41 educational meetings across the aforementioned clerkships, in collaboration with the course coordinators. After briefly clarifying the study, DB distributed URL links and QR codes, which gave students access to the questionnaire using their mobile devices. Before starting the questionnaire, participants signed an informed consent form.

### Instrument

We developed a questionnaire to explore students’ self-reported proficiency in SRL, their perceptions of the workplace learning context and its opportunities for learning and SRL, and students’ co-regulatory network characteristics (for a complete version of the questionnaire, see Additional file 1). For the present study, we only used the data on the composition of students’ co-regulatory networks (whom they engaged with to help regulate their learning) as well as on the purposes for which they engaged others in their co-regulatory networks. More specifically, we asked participants whom they would engage with when they wanted to discuss the regulation of their learning. Participants could select multiple responses from among the following eight options, hereinafter referred to as “actor groups” or “others”: peers, residents, physicians, workplace supervisor, nurses, mentor, friends, and family. For each actor group they selected, participants then indicated the specific focus of their interactions. They could select multiple responses from among five regulatory purposes, namely to discuss: 1) *learning goals;* 2) *learning strategies;* 3) *how to use suitable learning opportunities;* 4) *working on learning goals in the workplace;* and 5) *self-reflection and self-evaluation*. We based these response options on Zimmerman’s SRL model, which assumes that SRL processes and activities take place in three phases, that is, prior to a task (e.g., formulation of learning goals and strategic planning), during a task (e.g., how best to use learning opportunities, how to work on goals in specific settings), and following a task (self-reflection and self-evaluation) [21].

Before drafting the final questionnaire, we first pilot tested it on 10 respondents for comprehensibility of items, appropriateness of response options, and questionnaire length [22]. Following this process, we slightly modified several items to improve comprehensibility. Participants in our pilot tests were 5 medical students (enrolled in clinical clerkships at the time of this study), 2 residents (who had recently completed the undergraduate medical curriculum at the time of this study), 2 physicians and 1 psychologist). Participants were recruited using a snowball sampling strategy. Pilot tests revealed relevant others with whom students could potentially interact during clerkships. Moreover, we decided to measure the regulatory purposes at the level of actor groups rather than at the level of each individual within the network, as respondents indicated that the cognitive load necessary to complete the questionnaire was too high.

### Data analysis

For each actor group in students’ networks and for each regulatory activity, we calculated the total number of students indicating that they engaged in CRL with that particular actor group for that particular purpose. This allowed us to create an overview of the proportions of students who engaged the eight actor groups for each of the five regulatory activities. We calculated proportions for all students combined, as well as for each educational year. To examine whether first-, second-, and third-year students differed in the purposes for which they engaged others in their co-regulatory networks, we conducted analyses of variance (ANOVAs) comparing the proportions of students for each regulatory purpose across educational years. We corrected for multiple comparisons (Bonferroni correction). Because we were interested in trends across educational years, and less in demonstrating significance, we will present the results without correction and also give a description of the results with correction for multiple comparisons.

## Results

Of the 615 students we invited to participate, a total of 403 completed the questionnaire (65.5% response rate). These respondents included 145 (36%) first-year students, 142 (35%) second-year students, and 116 (29%) third-year students. The sample consisted of 284 women (70.5%) and 117 men, which is representative of the student population in the program (69% female). In presenting the results, we will first describe the aggregated data (i.e., for all students, irrespective of year of study). Subsequently, we will focus on the trends in students’ network deployment across educational years, zooming in on actor groups in students’ co-regulatory networks, the purposes of co-regulatory network deployment, and interaction between actor groups and purposes.

**Fig. 1:**
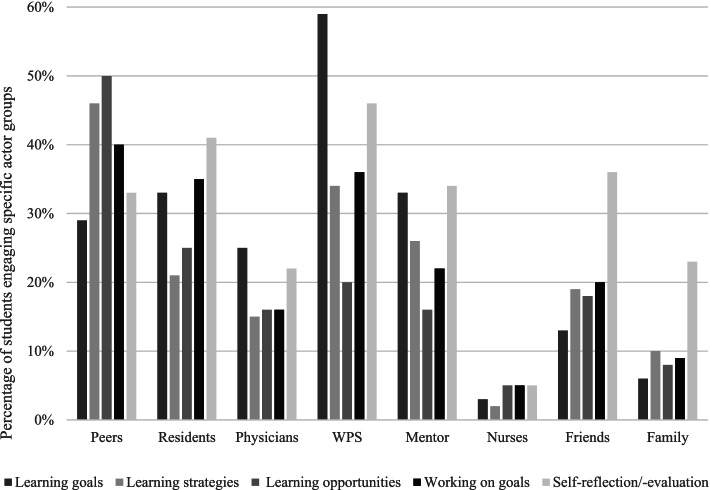
Distribution of co-regulatory purposes

Figure 1 gives an overview of the eight actor groups in students’ co-regulatory networks and the purposes for which all students, irrespective of educational year, engaged each group. Although students engaged all actor groups in their co-regulatory networks, they did so to varying degrees and for various purposes. Peers, workplace supervisors, and residents in particular seemed to figure prominently in students’ co-regulatory networks; After these groups, the actors that were engaged the most were, in descending order, mentors, friends, physicians, family, and nurses. Nurses were consistently engaged the least in students’ networks. The most frequently mentioned purpose of engaging others was to discuss self-reflections and self-evaluations, followed by learning goals, working on learning goals in the workplace, learning strategies, and how to use suitable learning opportunities. Students primarily involved workplace supervisors or mentors, for that matter, with the aim to discuss learning goals, self-reflections, and self-evaluations, whereas they engaged peers to discuss learning strategies, learning opportunities, and how to work on goals in the workplace. Friends and family were among the actor groups students less frequently called upon, and when they did so, interactions were mainly targeted at discussing self-reflections and self-evaluations. In fact, discussing self-reflections and self-evaluations was among the two purposes most frequently mentioned for all actor groups, the group of peers excepted.

**Fig. 2:**
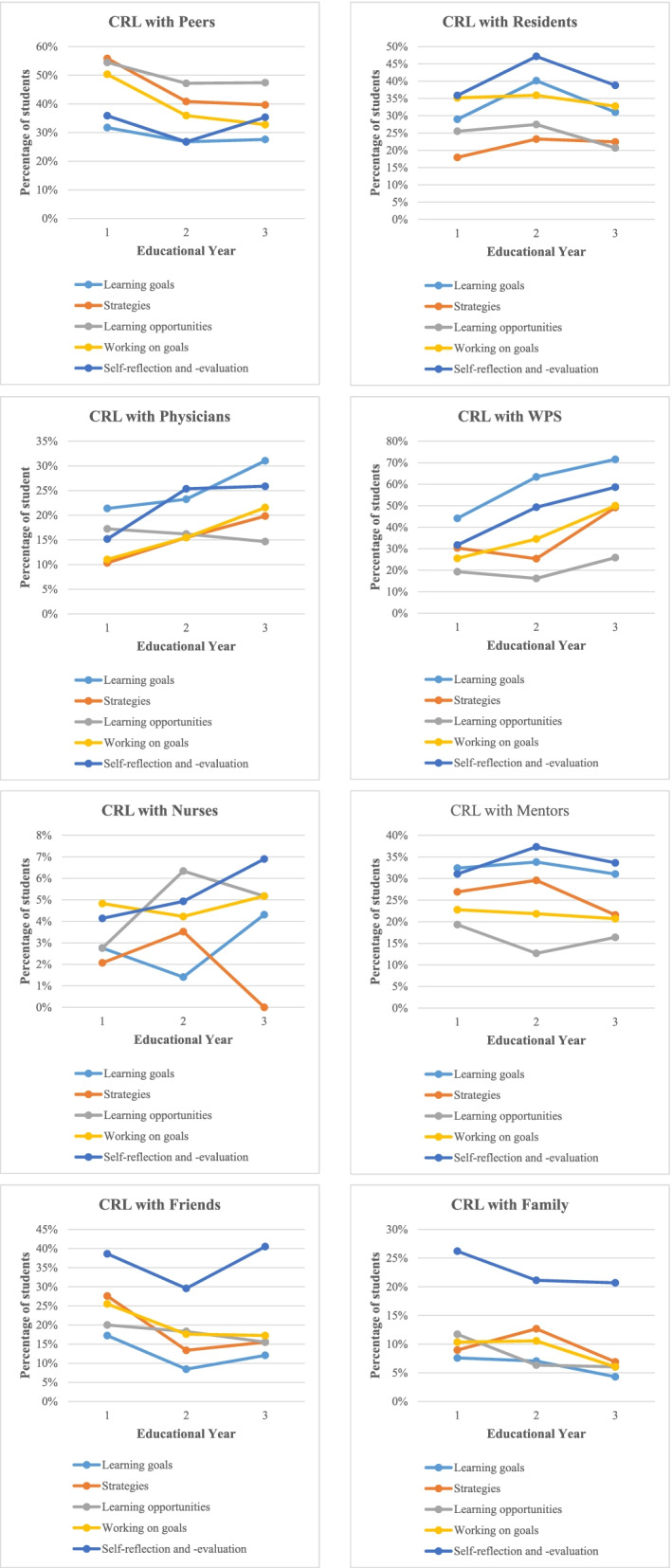
Distribution of co-regulatory purposes across educational years

Figure 2 and Supplementary Table 1 (Supplemental Online Material) present the distribution of students’ purposes of engaging the eight actor groups in their co-regulatory networks across educational years. As the percentages of students engaging their network differed greatly across actor groups, the Y-axes of the various graphs in Fig. 2 are presented on different scales. From these data, we may infer that students of all years actively engaged their peers, but especially so in their first year, and that, once more seasoned, they started to favor workplace supervisors. Figure 2 furthermore shows that co-regulation of learning strategies with nursing staff dropped to zero for third-year students. Table 1 presents significant findings from the ANOVA tests that compared first-, second-, and third-year students in terms of the purposes for which they engaged others in efforts to regulate their learning. Overall, we discerned two trends: across educational years, students increasingly engaged their workplace supervisors to discuss learning goals, learning strategies, self-reflections, and self-evaluations, and they less frequently called upon their peers to discuss learning strategies and how to work on learning goals in the workplace. Compared to second- and third-year students, first-year students were, moreover, more inclined to discuss learning strategies with friends. After we corrected for multiple comparisons (Bonferroni), the reduced tendency to discuss learning strategies with peers became non-significant, and the shift in discussing how to work on learning goals with peers became marginally significant (.007 with α = .0062).

**Table 1:** Results of the ANOVA tests comparing proportions of students across three educational years, (*N* = 403)

	Mean Y1	Mean Y2	Mean Y3	Mean difference (Y2-Y1)	Mean difference (Y3-Y1)	Mean difference (Y3-Y2)	F
**Discussing learning goals**							
WPS	0.44	0.63	0.72	0.19**	0.28***	0.09	11.48
**Discussing learning strategies**							
Peers	0.56	0.41	0.40	−0.15*	−0.16*	0.01	4.61
WPS	0.30	0.25	0.49	−0.05	0.19**	0.24***	9.05
Friends	0.28	0.13	0.16	−0.15**	− 0.12*	0.03	5.47
**Discussing working on learning goals**							
WPS	0.26	0.35	0.50	−0.09	0.24***	0.15*	8.79
Peers	0.50	0.36	0.33	−0.14*	− 0.17*	−0.03	5.07
**Discussing self-reflections / self-evaluations**							
WPS	0.32	0.49	0.59	0.17**	0.27***	0.10	10.42

Results of the ANOVA tests comparing the proportions of first-, second-, and third-year students regarding the purposes for which they engaged others in their co-regulatory networks. “Mean” refers to the proportion of all students (*N* = 403) who engaged the respective group in the said regulatory activity (discussing learning goals, learning strategies, how to work on learning goals, and self-reflections/self-evaluations). The degrees of freedom for the ANOVA tests were 2 and 400, respectively. For post-hoc tests we mentioned only the rotations that differed significantly following the pairwise t-test

* = *p* < 0.05, ** = *p* < 0.01, *** = *p* < 0.001

## Discussion

This study explored whom students in different phases of clinical medical training included in their co-regulatory networks as well as the purposes for which they engaged others in these networks. It is important to consider that such networks extend beyond the direct clinical workplace, with many actor groups playing a role in students’ efforts to regulate their learning. Our findings suggest that medical students select and engage others in their co-regulatory networks to varying degrees and for various purposes. Moreover, the actor groups they engage with and the purposes of engagement seem to shift over time. Subject to variations across individual students and study phases, students engaged all eight actor groups and covered all five regulatory purposes included in our study. Discussing self-reflections and self-evaluations were consistently among the purposes most frequently mentioned by students.

Our findings suggest that clerkship students purposefully engage others in their co-regulatory networks, with self-reflections and self-evaluations often being the focus of such interactions. This may seem surprising at first, as embedding reflection in medical education has repeatedly been shown to be difficult or even problematic [23, 24]. Prior research has demonstrated, for instance, that reflection does not occur as often as is desirable [24], or fairly often turns into a box-ticking exercise, with students telling others what they think they want to hear rather than engaging in true reflection [23]. The students in our study, however, actively discussed self-reflections and self-evaluations with others, which points to their willingness and ability to deploy others in their co-regulatory network for this particular purpose. In interpreting this finding, we must consider the context in which this study was situated: The Maastricht University medical program requires students to engage in self-evaluation and reflective writing throughout the program. Before entering clinical clerkships, students have spent three years developing relevant skills in longitudinal student-mentor relationships. This preparatory training may explain students’ ability to purposefully and meaningfully engage others in their networks to co-regulate self-reflections and self-evaluations. As such, our finding underscores the importance of curriculum design and support (e.g., training) in incentivizing students to engage others in reflection.

Another important finding is that students deliberately engaged clinical supervisors (i.e., workplace supervisors, physicians, and residents) in their regulatory learning. They often did so with the aim to discuss learning goals, self-reflections, and self-evaluations. This tendency, too, may be a product of the research setting: By expecting students to formulate learning goals and learning plans, discuss these with workplace supervisors and mentors, and jointly reflect on the process afterwards, the Maastricht curriculum drives CRL in such a way that it has largely permeated workplace learning [20]. As such, these curricular and assessment demands on student-clinical supervisor interactions hint at the presence of an extrinsic dimension, begging the question of how we can support students in shaping their regulation of learning in a more intrinsically motivated fashion.

A third finding worthy of note is that students extended their co-regulatory networks beyond the clinical workplace to include friends and family members. Interestingly, although they did not engage them as frequently as other actor groups, when they did engage them, the aim was mainly to discuss self-reflections and self-evaluations. A potential reason could be that students found in them a safe environment, which is an important condition for self-reflection [25, 26]. As their connection with family members and friends often stretches beyond professional settings and learning situations, students might have felt more comfortable having reflective and evaluative conversations with them. From a different angle, reflections and evaluations can differ in form and focus. This means that students may have wanted to engage different actor groups for different regulatory purposes. Consequently, they may have called upon their “personal” networks (i.e., friends and family) to discuss more personal matters, such as self-reflections on their identity formation and personal development rather than to evaluate task performance. These findings expose the need for medical education to realize that friends and family play an important role in students’ self-reflection and self-evaluation and to reconsider the extent to which we capitalize on the opportunities they offer. We might need to train students to engage in and elicit meaningful learning conversations not only with clinicians but also with friends and family.

We also found that students tend to increase engagement with workplace supervisors in co-regulatory networks and decrease engagement with peers and friends while progressing through the program. These findings echo previous research suggesting that the role of peers in CRL is particularly important at the start whereas more experienced others (residents and physicians) come to the fore toward the end of clinical education [5, 12]. A growing need to reflect on their professional identity [12] and on their future career path might induce more seasoned students to seek interactions with the people they consider their role models [27]. As these students, moreover, are more likely to understand the workplace dynamics of existing clinical communities of practice than novice students [28, 29], they may be more inclined to interact with experienced physicians within their networks. An important lesson to draw from our study is that any group of actors can play an important part in students’ SRL and CRL. As such, it is imperative that we support these different actors in fulfilling a meaningful role in co-regulating students’ learning processes and activities. To render CRL more effective, we must therefore make clinicians and residents aware of students’ specific regulatory purposes so that they can assist students in explicating these purposes.

Finally, we were able to discern a pattern of students underutilizing nurses. Research into the relationships between nurses and medical students has, indeed, suggested that their interactions can be of poor quality, impeding collaboration [30, 31]. Other studies, however, have argued that nurses do play and important, albeit small, role in helping create a safe learning environment and identifying learning opportunities [5]. While medical students tend to perceive nurses as more caring and less arrogant than physicians, they also regard them as less competent and having less status [32]. Yet other studies have added that medical students sometimes find it difficult to understand nurses’ professional and educational roles [33], thereby creating barriers for students to engage nurses in regulatory learning. Therefore, we must first and foremost develop a clear conception of the educational role that nurses can play in students’ regulation of learning.

Our results carry practical implications. Students might benefit from existing training programs focused on network building to [34] help them become aware of networking goals and benefits and improve their network-building skills [34]. Such trainings should focus on clarifying the potential roles of actor groups in students’ learning processes. Our findings also imply that faculty development programs should pay attention to clinical teachers’ roles in CRL and provide support in helping them fulfill these roles. To this end, they may offer clinical teachers a similar training on network building to raise awareness of their role and offer tools to help students regulate their learning. When the relevance of interactions and discussions within co-regulatory networks is made explicit, both students and supervisors might be better able to purposefully engage in meaningful co-regulatory interactions that help students to regulate their learning in clinical workplaces. Lastly, to help students effectively co-regulate their learning, SRL and CRL should be made explicit in both the curriculum and the assessment program.

### Limitations and future directions

This study has limitations. First, our conclusions were based on self-reported data. It may have been difficult for students to recall whom they engaged in their networks and the purposes for which they did so. In network studies, however, self-reports are widely used. Second, as we focused solely on interactions initiated by students, we were able to describe only part of students’ co-regulatory activities during clerkships. Third, although we derived the five response options representing regulatory purposes from SRL models *and* pilot testing, they constitute only part of the processes on the regulatory spectrum with a relatively low level of granularity. Through pilot testing, however, we aimed to minimize this limitation. The present study was among the first to explicitly focus on and explore co-regulatory networks to increase our understanding of medical students’ regulatory learning. Its limitations should be considered in light of this exploratory nature. Future research endeavors might want to move beyond self-reported data and the limitations they are subject to and take an ethnographic approach by observing how students interact with others to co-regulate their learning during clerkships. To cover the full scope of CRL, they might also want to focus on interactions initiated by others in the environment. Finally, we should consider qualitative social network studies that enable a more in-depth exploration and description of how regulatory processes and activities are embedded in co-regulatory networks.

## Conclusions

The findings from this study emphasize and reinforce the increasingly acknowledged notion within medical education that students’ regulation of learning is embedded in social interactions within co-regulatory networks. They do so by elucidating the extent to which regulatory purposes are distributed across students’ co-regulatory networks and exposing which processes students from different educational years regulate and with whom they do so. These insights open up new opportunities to embed learning from and with others in medical education to produce health professionals who are able to think and work beyond the self.

## Supplementary Information

**Additional file 1.** Self-Regulated Learning at Work Questionnaire.

**Additional file 2: Supplementary Table 1**. Percentages of first-, second-, and third-year students engaging others in their co-regulatory networks for five regulatory purposes.

## Data Availability

The datasets used and/or analysed during the current study are available from the corresponding author on reasonable request.
