# Supplementary material for: How medical students co-regulate their learning in clinical clerkships: a social network study
Source: BMC Med Educ. 2022 Mar 21;22:193. doi: 10.1186/s12909-022-03259-0 (PMC8939067; doi:10.1186/s12909-022-03259-0)
Supplement: Supplementary file 2 — Additional file 2: Supplementary Table 1. Percentages of first-, second-, and third-year students engaging others in their co-regulatory networks for five regulatory purposes. [file 12909_2022_3259_MOESM2_ESM.docx]

| **Supplementary Table 1.** Percentages of first-, second-, and third-year students engaging others in their co-regulatory networks for five regulatory purposes. | | | | | | |
| --- | --- | --- | --- | --- | --- | --- |
|  | **Year** | **Learning**  **goals** | **Learning strategies** | **Learning opportunities** | **Working on goals** | **Self-reflection and self-evaluation** |
| **Peers** | 1 | 32 | 56 | 54 | 50 | 36 |
|  | 2 | 27 | 41 | 47 | 36 | 27 |
|  | 3 | 28 | 40 | 47 | 33 | 35 |
| **Residents** | 1 | 29 | 18 | 26 | 35 | 36 |
|  | 2 | 40 | 23 | 27 | 36 | 47 |
|  | 3 | 31 | 22 | 21 | 33 | 39 |
| **Physicians** | 1 | 21 | 10 | 17 | 11 | 15 |
|  | 2 | 23 | 15 | 16 | 15 | 25 |
|  | 3 | 31 | 20 | 15 | 22 | 26 |
| **WPS** | 1 | 44 | 30 | 19 | 26 | 32 |
|  | 2 | 63 | 25 | 16 | 35 | 49 |
|  | 3 | 72 | 49 | 26 | 50 | 57 |
| **Mentor** | 1 | 32 | 27 | 19 | 23 | 31 |
|  | 2 | 34 | 30 | 13 | 22 | 37 |
|  | 3 | 31 | 22 | 16 | 21 | 34 |
| **Nurses** | 1 | 3 | 2 | 3 | 5 | 4 |
|  | 2 | 1 | 4 | 6 | 4 | 5 |
|  | 3 | 4 | 0 | 5 | 5 | 7 |
| **Friends** | 1 | 17 | 28 | 20 | 26 | 39 |
|  | 2 | 8 | 13 | 18 | 18 | 30 |
|  | 3 | 12 | 16 | 16 | 17 | 41 |
| **Family** | 1 | 8 | 9 | 12 | 10 | 26 |
|  | 2 | 7 | 13 | 6 | 11 | 21 |
|  | 3 | 4 | 7 | 6 | 6 | 21 |
| Percentages of first-, second-, and third-year students who engaged peers, residents, physicians, workplace supervisors (WPS), mentor, nurses, friends, and family for each of the five regulatory purposes, namely to discuss: learning goals; learning strategies; how use of suitable learning opportunities; working on learning goals in the workplace; and self-reflection and self-evaluation. | | | | | | |
